# Analysis of volatility characteristics of five jujube varieties in Xinjiang Province, China, by HS‐SPME‐GC/MS and E‐nose

**DOI:** 10.1002/fsn3.2607

**Published:** 2021-09-29

**Authors:** Yuxing Liu, Yueying Sang, Jingyu Guo, Weida Zhang, Tianyu Zhang, Hai Wang, Shaobo Cheng, Guogang Chen

**Affiliations:** ^1^ School of Food Science and Technology Shihezi University Shihezi China; ^2^ Academy of Agricultural Planning and Engineering Beijing China

**Keywords:** E‐nose, HS‐SPME‐GC/MS, jujube, multivariate analysis, volatile components

## Abstract

In this study, headspace solid‐phase microextraction coupled with gas chromatography‐mass spectrometry (HS‐SPME‐GC/MS) was used to identify individual volatile compounds in five jujube varieties, and E‐nose was used to identify their flavor. The results showed that a total of 45 volatile compounds were detected by GC‐MS in the five varieties, and the proportion of acids was the highest (38.29%–54.95%), followed by that of aldehydes (22.94%–47.93%) and esters (6.33%–26.61%). Moreover, different varieties had obviously different volatile components. E‐nose analysis showed that the R7 and R9 sensors were more sensitive to the aroma of jujube than other sensors. The strong response of R7 sensor was attributed to terpenes (or structurally similar substances) in jujube fruit, such as 1‐penten‐3‐one, 2‐octenal, (E)‐2‐heptanaldehyde, and (E)‐2‐hexenal and that of R9 sensor was attributed to the cyclic volatile components such as benzaldehyde, benzoic acid, and methyl benzoate. The multivariate data analysis (PCA, OPLS‐DA, and HCA) of the results of GC/MS and E‐nose showed that the five varieties could be divided into three groups: (1) *Ziziphus jujuba* Mill. cv. Huizao (HZ) and *Z. jujuba* cv. Junzao (JZ). Acids were the main volatile components for this group (accounting for 47.44% and 54.95%, respectively); (2) *Z. jujuba* cv. Hamidazao (HMDZ). This group had the most abundant volatile components (41), and the concentrations were also the highest (1285.43 µg/kg); (3) Winter jujube 1 (*Z*. *jujuba* cv. Dongzao, WJ1) and Winter jujube 2 (*Z*. *jujuba* cv. Dongzao, WJ2). The proportion of acids (38.38% and 38.29%) and aldehydes (40.35% and 38.19%) were similar in the two varieties. Therefore, the combination of headspace solid‐phase microextraction coupled with gas chromatography‐mass spectrometry and E‐nose could quickly and accurately identify the volatile components in jujube varieties from macro‐ and microperspectives. This study can provide guidance for the evaluation and distinguishing of jujube varieties and jujube cultivation and processing.

## Graphical abstract

The volatile components of fruits of five jujube varieties cultivated in Xinjiang Province, China, were analyzed using the HS‐SPME‐GC/MS and E‐nose. GC‐MS and E‐nose results were separated by multivariate analysis. This study provides guidance for the selection of raw materials and jujubes processing.

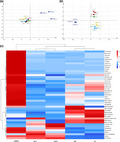

## 1 INTRODUCTION

Jujube (*Ziziphus jujuba* Mill.), a member of Rhamnaceae family, originates from the Yellow River basin in China. It has been cultivated for more than 4000 years (Zhang et al., 2018). Jujube fruit is favored by many consumers for the abundance of components such as triterpenoids, phenolic acids, flavonoids, and polyphenols that have anti‐inflammatory, antiallergic, and antioxidant effects (Cheng et al., 2020; Jiang et al., 2019). Moreover, jujube products, such as fruit wines and jam, are considered healthy foods that are increasingly preferred by consumers around the world (Wojdylo et al., 2016). The aroma of jujube gives a special flavor characteristic to jujube products. However, most researches focus on the preservation and processing of jujube fruit (Cheng et al., 2020), while few studies focus on the volatile components in jujube fruit.

Volatile components are the secondary metabolites of fruit. The release of volatile components brings unique flavor profile for fruit (Janzantti & Monteiro, 2014). Therefore, volatile components can be used to evaluate the fruit quality (Liu, Du, et al., 2019). However, the volatile components may significantly vary in varieties (Barros‐Castillo et al., 2021; Chen et al., 2020), and the volatile components of a variety may also vary in production areas (Li et al., 2013; Spizzirri et al., 2019). Therefore, a comprehensive exploration of the volatile components of jujube fruit is essential, which is helpful for the raw material cultivation and selection for fruit processing industry (Wang, Wang, Deng, et al., 2019).

Headspace solid‐phase microextraction (HS‐SPME) is commonly used to extract the volatile components from fruit using fused silica fibers. Compared with simultaneous distillation extraction and volatile oil extraction, HS‐SPME can avoid thermochemical influence on volatile components during the extraction process. Moreover, the headspace solid‐phase microextraction coupled with gas chromatography‐mass spectrometry (HS‐SPME‐GC/MS) provides a rapid and accurate tool for qualitative and quantitative analysis of the volatile components in fruit (Chen et al., 2020). Galindo et al. (2015) used this method to detect the volatile components in jujube fruit, finding that there were a total of 18 volatile components detected, and the aldehydes and acids such as hexanal, (E)‐2‐hexanal, benzaldehyde, hexanoic acid, and decanoic acid accounted for 95%. Wang, et al. (2019) also used this method to detect the volatile components in jujube fruit, finding that there was significant difference in volatile components among different varieties.

The electronic nose (E‐nose) is a bionic equipment with multiple chemical sensors that can accurately evaluate the volatility characteristics of fruit (Yang et al., 2016). The combination of HS‐SPME‐GC/MS and E‐nose can analyze the volatile components of fruit from macroscopic and microscopic perspectives. Currently, this method has been widely used for shelf‐life assessment (Wang, Baldwin, et al., 2019; Yang et al., 2020), freshness assessment (Dou et al., 2020; Pennazza et al., 2013), and fruit processing monitoring (Xu et al., 2019; Yang et al., 2016). Chen et al. (2018) used this method to analyze the volatile components in jujube fruit, finding that there were significant differences in the volatility characteristics among different varieties.

Xinjiang Uygur Autonomous Region of China is the main production area of jujube in the world, but the volatile components of different jujube varieties in Xinjiang have not been determined. Therefore, in this study, five jujube varieties mainly cultivated in this area were used as test materials, to identify the volatile components using HS‐SPME‐GC/MS and E‐nose, and the differences in volatile components among different varieties were determined by using multivariate data analysis. This study contributes to the breeding of excellent jujube varieties and the fruit processing industry.

## 2 MATERIALS AND METHODS

### 2.1 Sample preparation

Jujube fruit of five varieties was collected from Xinjiang Uygur Autonomous Region, China, in September 2019. Among them, *Z. jujuba* cv. Huizao (HZ) and *Z. jujuba* cv. Junzao (JZ) were collected from Kashgar (75°56′36″N, 39°23′48″E) and Aksu (80°20′32″N, 41°11′42″E), respectively. The planting area of the two varieties is the largest, and the yield is the highest in Xinjiang. *Z. jujuba* cv. Hamidazao (HMDZ), a native jujube variety, were collected from Hami (93°35′53″N, 43°12′48″). Winter jujube 1 (*Z*. *jujuba* cv. Dongzao, WJ1) and Winter jujube 2 (*Z*. *jujuba* cv. Dongzao, WJ2) were collected from Aksu (80°27′28″N, 41°8′39″E) and Kashgar (75°49′51″N, 39°30′21″E), respectively. Winter jujube is a new introduced variety in recent years. Fresh winter jujube is widely preferred by consumers. The fruit with similar size, uniform color, and no mechanical damages was collected by hand and delivered to the laboratory immediately. Fruit samples matured naturally (4–6 days) to the semired stage (the color of 50% of fruit surface became red) at room temperature of 25 ± 3℃, with relative humidity of 40 ± 5%. Then, the fruit of each variety (1 kg) was peeled, stoned, cut into small pieces (3 mm in thickness), frozen in liquid nitrogen, and stored at −80℃.

### 2.2 HS‐SPME extraction of volatile components

The volatile components of jujube fruit were extracted and determined according to the method proposed by Song et al. (2019), using an aged SPME extraction head (50/30 μm PDMS/CAR/DVB). Fruit sample (4 g) was equilibrated in a 20 ml sample vial at 50℃ for 30 min, and the temperature of the solid‐phase microextraction apparatus was set at 50℃. After that, vials were stirred at 300 rpm and headspace extraction was performed for 30 min.

### 2.3 Quantification of volatile components by GC/MS

The extracted volatile components were analyzed with GC/MS (SQ‐456‐GC‐MS, Scion), and a DB‐WAX (30.0 m × 250 μm, 0.25 μm) chromatographic column was used. The temperature of the column incubator started at 40℃. After 3 min, the temperature increased to 100℃ at 6℃ /min. Then, it increased to 230℃ at 10℃/min and lasted for 6 min. The temperature of sample inlet was 200℃, and the temperature of detector was 250℃. The carrier gas was N_2_ at a flow rate of 0.8 ml/min. The MS cleavage was performed using electron ionization mode with an electron energy of 70 eV. The temperature of ion source was 200℃. The interface temperature was 250℃. The mass was in the range of 33–400 u.

### 2.4 E‐nose analysis of the volatility characteristic of jujube fruit

E‐nose analysis was performed with the method of Chen et al. (2018). Fruit samples were crushed and sieved through a 60 mesh. After that, the powder (3.0 g) was transferred in 15‐ml headspace vial, sealed, and equilibrated at 30℃ for 30 min. After equilibration, the samples were placed in an E‐nose device equipped with 10 sensors (PEN3, AirSense) for detection, and the responses in 1 min were recorded.

### 2.5 Data analyses

Data were analyzed using SPSS 18.0 (IBM). The concentrations of volatile components were expressed as mean ±standard (standard deviation). Duncan's test was used to detect the significant difference in the data of GC/MS and E‐nose analysis for different varieties. GC/MS and E‐nose data were analyzed using PCA, OPLS‐DA, and HCA, and plotting was performed using Origin 2018 (Origin Lab Co.).

## 3 RESULTS AND DISCUSSION

### 3.1 HS‐SPME‐GC/MS analysis of volatile components of jujube fruits

#### 3.1.1 Qualitative and quantitative analysis of volatile components

A total of 45 volatile components were detected in jujube fruit (Table 1), including acids (14), aldehydes (14), esters (10), ketones (5), and alcohols (2). Acids and aldehydes were the main volatile components in jujube fruit, accounting for more than 70%. Saturated fatty acids such as hexanoic acid (40.44–91.88 µg/kg), lauric acid (14.42–77.88 µg/kg), and decanoic acid (10.94–85.39 µg/kg) were the main sources of jujube aroma. The concentration of acetic acid (the source of tart flavor of fruit) in HZ, HMDZ, and JZ (48.136–138.044 µg/kg) was significantly higher than that in WJ1 and WJ2. Aldehydes including hexanal (4.34–53.65 µg/kg), (E)‐2‐hexenal (11.31–131.43 µg/kg), (E)‐2‐heptenal (10.24–30.76 µg/kg), 2‐octenal (14.44–114.52 µg/kg), and benzaldehyde (5.56–239.26 µg/kg) contributed to the green aroma of jujube fruit. Esters, mainly including methyl hexanoate (7.97–73.59 µg/kg), methyl decanoate (3.86–11.92 µg/kg), and methyl laurate (4.02–12.54 µg/kg), accounted for 6.67%–26.24%. They were mainly generated from the primary metabolite, alcohols, ethanol dehydrogenase, and ethanol aminotransferase (AAT) in jujube fruit and contributed Fruity aroma (Pott et al., 2019). In addition, 1‐penten‐3‐one, 3‐octanone, formic acid, lauric acid, palmitic acid, oleic acid, 2‐octenal, (Z)‐6‐nonenal, and 3‐octanol, which were not reported in previous studies, were found for the first time in our study (Chen et al., 2018; Song et al., 2020). These volatile components may be related to the growth environment and jujube variety (Liu et al., 2018).

**TABLE 1:** The content of volatile compound in different cultivars of jujube (µg/kg)

	Compound^a^	CAS	RT^b^	Different varieties of jujube					Aroma description ^c^
				HZ	HMDZ	JZ	DZ1	DZ2	
Ester									
1	Ethyl acetate	141‐78‐6	3.497	1.5465 ± 0.1851	—	2.0489 ± 0.373	—	—	Fruity
2	Methyl valerate	624‐24‐8	7.473	2.1662 ± 0.2174	8.114 ± 4.7921	0.9634 ± 0.266	1.2099 ± 0.1805	—	Sweet, Green Fruity
3	Methyl hexanoate	106‐70‐7	9.698	73.5927 ± 5.9116	46.384 ± 3.5741	7.972 ± 2.812	23.7444 ± 2.1484	31.3131 ± 5.7803	Fruity
4	Ethyl hexanoate	123‐66‐0	10.641	2.3794 ± 0.6065	—	0.6639 ± 0.2946	—	—	Fruity
5	Methyl decanoate	110‐42‐9	17.812	10.2243 ± 0.9485	7.6115 ± 2.0889	14.9179 ± 6.0995	3.8628 ± 0.8979	4.5011 ± 0.6899	Wine
6	Methyl benzoate	93‐58‐3	18.312	—	7.5927 ± 1.2076	1.4581 ± 0.1954	—	1.0467 ± 0.4402	Green
7	Ethyl caprate	110‐38‐3	18.409	3.0755 ± 0.3734	3.2558 ± 0.4879	9.8323 ± 2.278	—	—	Sweet
8	Methyl laurate	110‐38‐3	20.471	10.6552 ± 1.8072	6.0783 ± 0.7732	12.5378 ± 5.1984	6.9085 ± 2.1444	4.0214 ± 0.2354	Fatty
9	Methyl myristate	111‐82‐0	23.156	5.936 ± 1.2003	3.448 ± 0.2697	6.4695 ± 3.0147	5.2066 ± 1.7087	2.9671 ± 0.0534	Fatty
10	Methyl palmitate	1120‐25‐8	25.073	3.8114 ± 0.8201	1.7779 ± 0.4385	4.1427 ± 2.3588	1.1425 ± 1.8816	1.9872 ± 0.2507	Fatty
	Total			116.0466 ± 12.2815	85.7672 ± 13.7348	62.6503 ± 23.6533	44.6037 ± 9.5296	45.8366 ± 7.4499	
Ketone									
11	1‐Pentene−3‐one	1629‐58‐9	5.934	—	—	—	7.2605 ± 2.0159	14.5742 ± 2.097	—
12	3‐Octanone	106‐68‐3	11.436	—	8.7203 ± 2.6884	—	—	—	Nut
13	1‐Octen−3‐one	4312‐99‐6	12.477	3.4631 ± 1.3882	5.6316 ± 0.6684	3.842 ± 1.9852	0.797 ± 0.3395	1.3922 ± 1.0546	Mushroom
14	Acetone	513‐86‐0	12.526	8.5259 ± 0.8239	20.8164 ± 6.5911	4.5386 ± 0.8666	—	—	—
15	6‐Methyl−5‐hepten−2‐one	110‐93‐0	13.423	2.9769 ± 0.8869	1.8557 ± 0.6098	3.0098 ± 0.6857	3.4359 ± 0.6584	2.1835 ± 1.4328	‐
	Total			14.9659 ± 3.099	37.024 ± 10.5577	11.3904 ± 3.5375	11.4934 ± 3.0138	18.1499 ± 4.5844	
Acid									
16	Formic acid	64‐18‐6	15.582	—	17.1723 ± 15.2323	1.7127 ± 1.4867	1.3325 ± 0.4333	—	Vinegar
17	Acetic acid	64‐19‐7	15.65	55.5279 ± 4.7921	138.0447 ± 72.6354	48.1361 ± 10.4348	3.5781 ± 0.638	6.0841 ± 1.2914	Vinegar
18	Valeric acid	109‐52‐4	19.761	3.277 ± 0.6526	7.2822 ± 1.8265	0.3621 ± 0.1553	—	—	—
19	Hexanoic acid	142‐62‐1	21.005	58.1909 ± 7.1156	91.8843 ± 24.237	40.4467 ± 12.7766	47.8768 ± 5.691	50.0631 ± 9.0001	Rancid, Fatty
20	Heptanoic acid	111‐14‐8	22.152	1.3363 ± 0.2128	24.6731 ± 6.2289	1.9742 ± 0.203	2.3677 ± 0.4925	1.6212 ± 0.4636	Rancid, Fatty
21	Octanoic acid	124‐07‐2	23.255	5.5912 ± 0.9081	40.882 ± 9.9832	6.0903 ± 0.5583	4.92 ± 1.0963	4.7419 ± 1.4665	Fatty, Rancid
22	Nonanoic acid	112‐05‐0	24.292		3.3556 ± 0.2551		1.5956 ± 0.3533	1.1633 ± 0.4033	Cheese
23	Lauric acid	143‐07‐7	25.261	32.192 ± 4.2684	77.8844 ± 18.4299	54.7865 ± 3.0763	21.0426 ± 4.8977	14.4281 ± 2.9078	Metal
24	Decanoic acid	334‐48‐5	25.273	32.3811 ± 3.8804	85.3972 ± 24.6526	59.3526 ± 4.5013	10.9419 ± 2.9719	17.6049 ± 4.8728	Fatty, Fruit
25	Palmitic acid	57‐10‐3	25.918	3.1494 ± 1.3416	13.7424 ± 0.9315	6.0291 ± 1.7683	3.9019 ± 1.8972	1.7385 ± 0.3663	Fatty
26	Oleic acid	112‐80‐1	26.218	1.2633 ± 0.7477	4.5813 ± 2.2796	2.6519 ± 1.9771	2.2375 ± 1.9046	—	Fatty
27	Benzoic acid	65‐85‐0	26.775	—	3.6273 ± 0.852	1.3807 ± 0.3484	—	—	—
28	Myristic acid	57677‐52‐8	30.061	2.4655 ± 0.3184	14.2238 ± 4.8593	3.4908 ± 0.5448	3.7171 ± 1.688	1.8935 ± 1.0318	—
29	Myrustoleic acid	544‐64‐9	30.928	11.5316 ± 1.4951	24.4197 ± 2.4477	15.1888 ± 2.6614	7.4869 ± 2.2023	4.8486 ± 0.1799	—
	Total			206.9062 ± 25.7328	547.1703 ± 184.851	251.1557 ± 37.7157	110.9986 ± 24.2661	104.1872 ± 21.9835	
Aldehyde									
30	Valeraldehyde	110‐62‐3	5.041	—	11.481 ± 4.7316	2.1828 ± 0.4671	3.9132 ± 1.7532	8.2771 ± 1.4105	Fruity
31	Hexanal	66‐25‐1	7.351	6.7221 ± 2.0754	51.6957 ± 14.4947	4.342 ± 1.8617	53.6532 ± 7.7393	47.977 ± 14.542	Green
32	3‐Hexenal	6789‐80‐6	8.638	—	—	—	0.9818 ± 0.4102	3.3142 ± 1.7421	Green
33	Heptaldehyde	111‐71‐7	9.668	2.2651 ± 0.7221	9.6866 ± 3.4574	2.0282 ± 0.6403	—	—	Fatty
34	(E)−2‐hexenal	6728‐26‐3	10.25	12.8755 ± 4.0738	95.1967 ± 21.3652	11.3098 ± 6.8174	117.8711 ± 8.3656	131.4309 ± 12.3358	Green, Leaf
35	Octanal	124‐13‐0	12.034	2.7221 ± 0.5506	23.1362 ± 8.3606	1.9826 ± 0.5698	—	1.5322 ± 0.4573	Fatty, Green
36	(E)−2‐Heptenal	18829‐55‐5	13.1	12.892 ± 4.5674	30.7253 ± 5.9987	15.1381 ± 4.7088	10.2388 ± 4.6894	12.9878 ± 6.9205	Fruity, Green
37	1‐Nonanal	124‐19‐6	14.411	1.5767 ± 0.2759	12.6763 ± 5.5609	2.0414 ± 0.2139	2.1594 ± 0.5061	2.0092 ± 1.0425	Fatty, Green
38	2‐Octenal	2363‐89‐5	15.312	19.7005 ± 6.3318	114.5214 ± 23.9052	24.7449 ± 7.6582	20.4357 ± 4.6491	14.1437 ± 7.1104	Spicy
39	(E,E)−2,4‐Heptadienal	4313‐03‐5	15.958	1.105 ± 0.2517	1.2707 ± 0.3504	—	3.4886 ± 2.1427	2.1347 ± 1.2128	Green,
40	Decanal	112‐31‐2	16.33	0.8973 ± 0.1401	2.9099 ± 0.1097	1.2197 ± 0.2001	2.4685 ± 0.7914	1.5091 ± 0.1797	Green
41	Benzaldehyde	100‐52‐7	16.887	35.8837 ± 7.8617	239.2583 ± 64.3818	64.4653 ± 4.9838	5.5644 ± 2.1812	8.184 ± 2.4702	Fruity
42	(Z)−6‐nonenal	2277‐19‐2	17.051	1.6642 ± 0.5017	5.9745 ± 2.9156	0.9872 ± 0.2838	1.5938 ± 0.594	1.8399 ± 1.0056	Green
43	(E)−2‐decenal	3913‐81‐3	18.53	1.7665 ± 0.6049	17.9033 ± 5.1645	2.6548 ± 0.5418	0.6724 ± 0.1384	—	—
	Total			100.0707 ± 27.9571	616.4359 ± 139.4311	133.0968 ± 28.9467	220.9119 ± 32.967	235.3398 ± 38.0936	
Alcohol									
44	3‐Octanol	589‐98‐0	14.781	—	3.5258 ± 0.7367	—	—	—	—
45	1‐Octen−3‐ol	3391‐86‐4	15.765	4.0812 ± 2.1232	70.7679 ± 21.0486	—	—	—	Mushroom
	Total			4.0812 ± 2.1232	73.9915 ± 22.325				
	Total			436.1072 ± 102.2497	1285.4348 ± 505.6561	457.0677 ± 124.8507	388.0076 ± 105.3241	403.5135 ± 114.7894	

Note

Data are presented as the mean ± SD (*n* = 3).

Abbreviations: HMDZ, Hamidazao; HZ, Huizao; JZ, Junzao.

^a^ Volatile compounds detected were integrated with the GC‐MS automatic deconvolution system and compared with the standard mass spectrum in the NIST 14 library. Every category volatile compounds are listed in order of retention time.

^b^ RT, the retention time (min) of identified compounds on capillary column DB‐WAX.

^c^ Odor descriptions were adapted from the online database (http://www.thegoodscentscompany.com).

**FIGURE 1:**
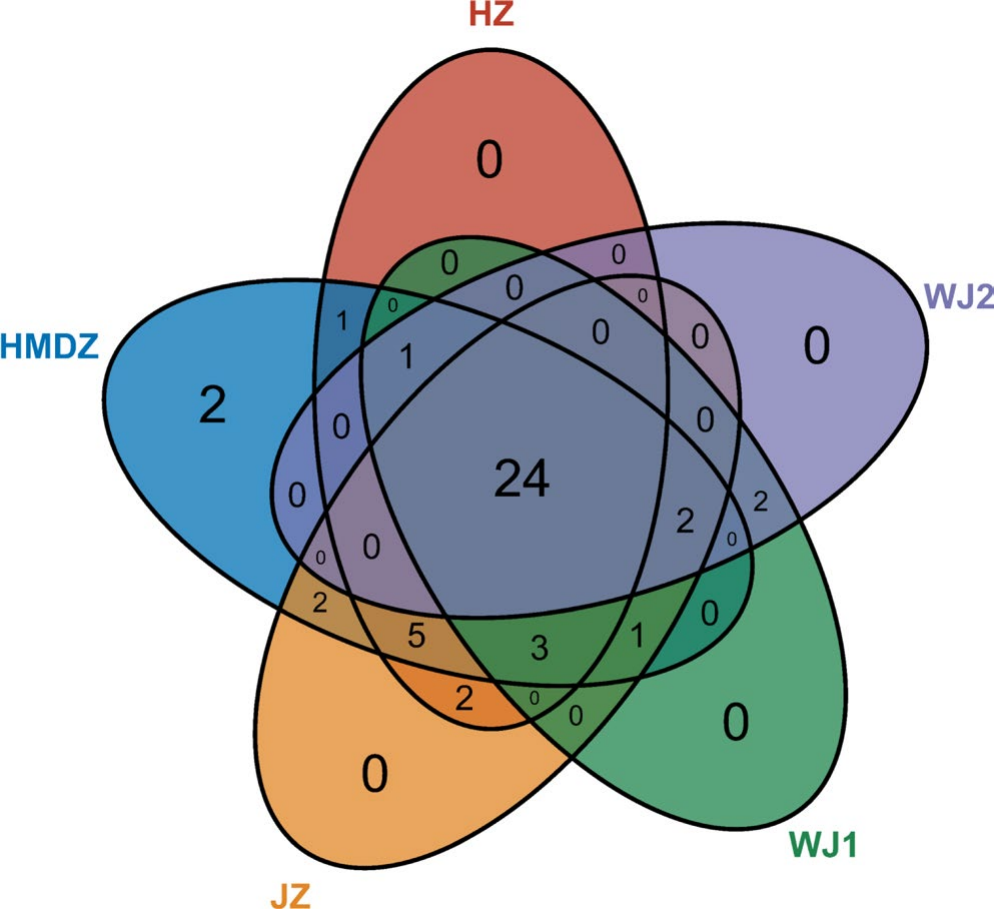
Venn diagram of volatile components in the fruit of different jujube varieties analyzed by HS‐SPME‐GC/MS

In this study, 36, 41, 37, 33, and 31 kinds of volatile components were identified in HZ, HMDZ, JZ, WJ1, and WJ2, respectively, of which 24 kinds of components were found in the five varieties (Figure 1 and Table 1). HMDZ had the most abundant volatile components (41) among the five varieties, and the concentration reached 1285.43 μg/kg. Moreover, 3‐octanol and 3‐octanone derivatives were only found in HMDZ, which were derived from octanoic acid (40.88 μg/kg) in HMDZ. HMDZ contained a large amount of volatile benzene derivatives, such as benzaldehyde (239.25 μg/kg), benzoic acid (3.62 μg/kg), and methyl benzoate (7.59 μg/kg), which might be due to the volatile substances produced by the side chain reaction of phenylalanine under the action of phenylalanine ammonia lyase in the shikimic acid pathway (Fock‐Bastide et al., 2014; Martin et al., 2016). A total of 37 kinds of volatile components were identified in JZ, and the concentration was 457.06 μg/kg, second to HMDZ. Acids were the main volatile components in JZ, accounting for 54.95% of the total volatile components. HZ had the highest concentration of esters (20.86%) among the five varieties. High concentration of methyl hexanoate (73.59 μg/kg) made JZ significantly different from other varieties and contributed greatly to the aroma of JZ. WJ1 and WJ2 were the same variety from different production areas. GC/MS results showed that they had similar volatile components (33 and 31 kinds) and concentrations (388.01 and 403.51 μg/kg). Moreover, the high concentration of hexanal (47.98–53.65 μg/kg) and (E)‐2‐hexanal (117.87–131.43 μg/kg) contributed greatly to the Green and Leaf aroma. Surprisingly, 1‐penten‐3‐one and 3‐hexenal were the volatile components only found in WJ1 and WJ2.

The GC/MS analysis showed that acids were the main volatile components in HZ and JZ, accounting for 47.44% and 54.95%, respectively, and aldehydes accounted for 22.95% and 29.12%, respectively. The proportions of acids and aldehydes in HMDZ (accounting for 45.97% and 47.93%, respectively), WJ1 (accounting for 38.38% and 40.35%, respectively), and WJ2 (accounting for 38.29% and 38.19%, respectively) were similar. Volatile acids are generated passively during plant growth or accumulated due to the amino acids under the action of amino acid dehydrogenase and amino acid transferase (Pott et al., 2019), while aldehydes are mostly generated from the oxidation of unsaturated fatty acids (Kostyra et al., 2021). The acids and aldehydes might be regulated by the content of amino acids and fatty acids in jujube fruit. This result differs from the reports of Wang et al. (2018) and Song et al. (2019). Their results showed that aldehydes were the most important volatile components in jujube fruit, accounting for more than 50%. It seems that there are more acids in jujube fruit cultivated in Xinjiang, China, which may be attributed to the growth environment.

#### 3.1.2 Multivariate data analysis by GC/MS

**FIGURE 2:**
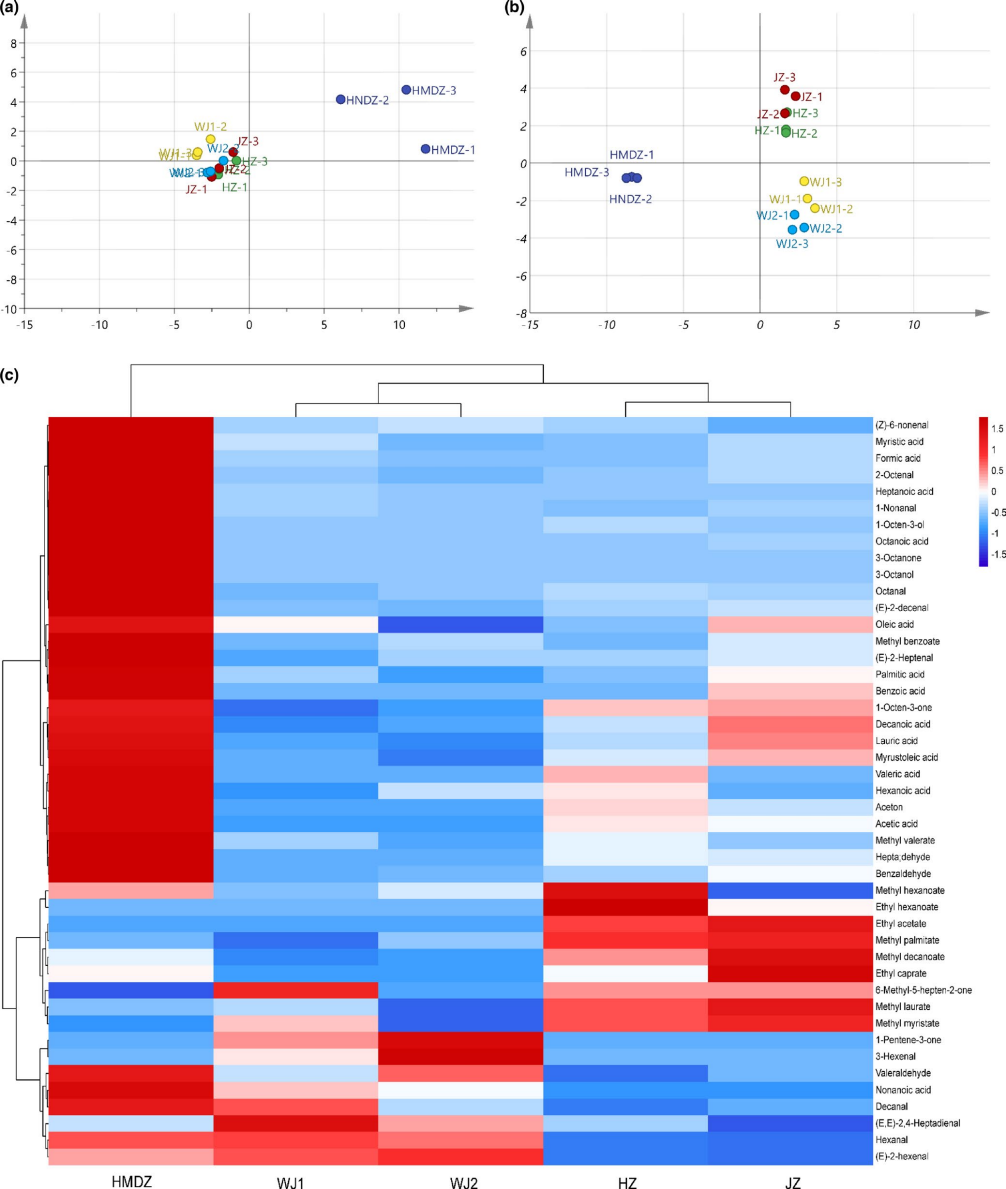
Volatile components in the fruit of different jujube varieties. (a) PCA analysis of the volatile components; (b) OPLS‐DA analysis of the volatile components; (c) HCA analysis of the volatile components

PCA analysis can reduce the dimension of the original data and retain the variability to separate samples (Khalil et al., 2017). The PCA results showed that PC1 and PC2 could explain 49.04% and 15.42% of the total variation, respectively (Figure 2a). The varieties, except for HMDZ, had overlapping areas, indicating that clear distinction did not exist. Therefore, to maximize the separation among samples, OPLS‐DA (a supervised classification method) was used to remove the variation of unrelated variables. After cross validation, the explanatory bias (R^2^Y) and predictive power (Q^2^Y) of the model were 0.975 and 0.895, respectively, which indicated that the model could distinguish the five samples well. According to the results of OPLS‐DA analysis (Figure 2b), the samples with inter group differences were divided into three groups: (1) HMDZ, (2) HZ and JZ, and (3) WJ1 and WJ2. According to the analysis of variable importance in projection (VIP), 12 volatile components (methyl caproate, decanal, methyl heptenone, methyl laurate, methyl myristate, methyl decanoate, myristic acid, (E)‐2‐hexenal, hexanal, palmitic acid, 2,4‐heptadienal, and 1‐octen‐one) with VIP >1 and *p* < .05 were considered as the main causes of sample separation (Liu, Deng, et al., 2019). HMDZ was mainly related to horizontal axis. It contained the most abundant volatile components (41) and had the highest concentration (1285.43 µg/kg), which might be the reason why it was separated alone. The kinds (37 and 36) and concentrations (436.10 and 457.06 μg/kg) of volatile components in HZ and JZ were similar, and the concentrations of many substances, such as acetic acid (55.52 and 48.14 μg/kg), hexanoic acid (58.19 and 40.44 μg/kg), and (E)‐2‐hexenal (12.87 and 11.39 μg/kg), were also similar. Therefore, both of them were in the same quadrant in OPLS‐DA analysis and positively correlated with horizontal axis. According to the factor loading, the most important volatile components were (E)‐2‐hexenal, methyl laurate, methyl myristate, methyl palmitate, and methyl heptenone. WJ1 and WJ2, the same variety of jujube from different areas, formed the third group because of their similar volatile components and concentrations. Moreover, our result showed that variety had a more significant influence on volatile components than production area, which was consistent with the results of Liu et al. (2018), Lukić et al. (2019), and Zhu et al. (2020). To verify the results, HCA analysis was performed using the squared Euclidean distance method (Wang, Wang, Deng, et al., 2019). The heat map shows the distribution of main volatile components of jujube fruit (Figure 2c). Similar to the distribution in OPLS‐DA analysis, the quantitative results were also divided into three groups by hierarchical clustering: (1) HMDZ, (2) HZ and JZ, and (3) WJ1 and WJ2, which verified the accuracy of previous multivariate analysis.

### 3.2 Analysis of volatile components in jujube fruits by E‐nose

#### 3.2.1 E‐nose analysis

**FIGURE 3:**
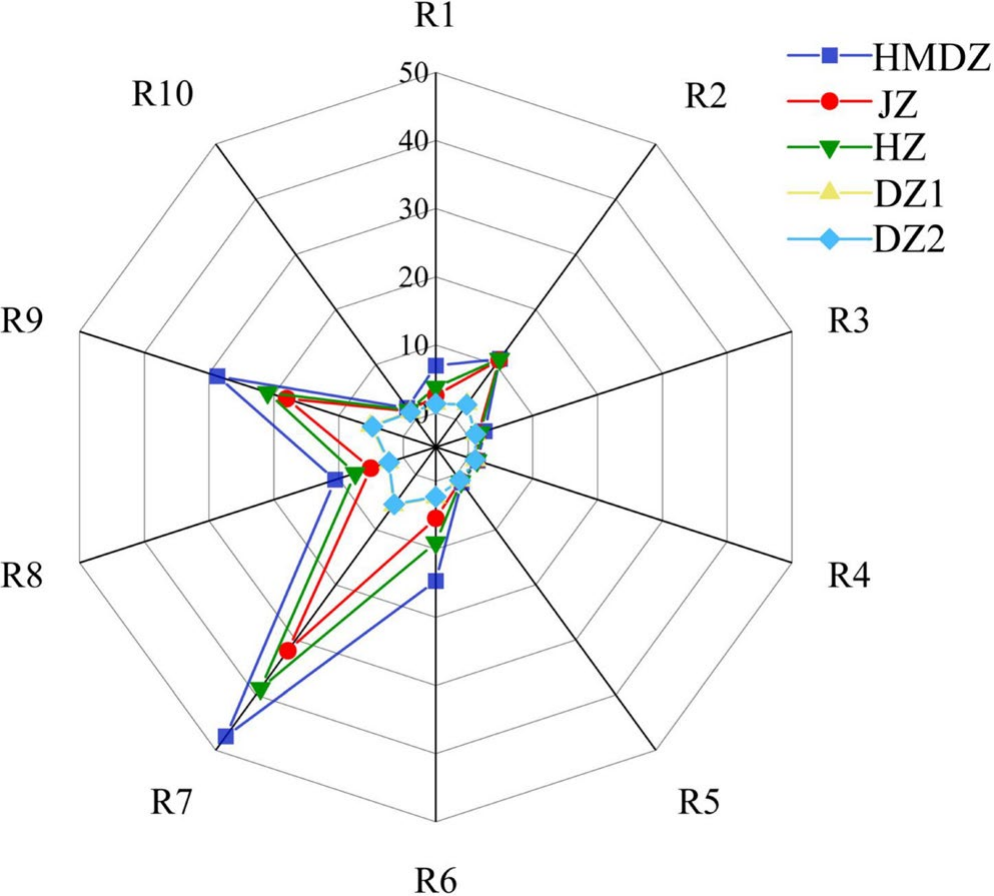
Radar map of E‐nose responses to the volatile components of different jujube varieties

The E‐nose is very sensitive to fruit aroma. A slight change in the aroma can be detected by the sensor (Yang et al., 2016). The E‐nose equipped with 10 metal oxide semiconductors (sensors) was used to analyze fruit aroma in this study. Among the sensors, only R2 (broadly sensitive), R6 (sensitive to methane), R7 (sensitive to terpenes and sulfides), R8 (sensitive to alcohols, aldehydes, and ketones), and R9 (sensitive to aromatics and organic sulfides) responded. The response value of R3 (ammonia, sensitive to aromatic components), R4 (mainly sensitive to hydrogen), R5 (sensitive to alkanes, aromatics, and small polar compounds), and R10 (sensitive to high concentration aliphatic compounds) were about 1 (Figure 3), which meant almost no response. The response value of R7, R9, R6, and R2 was 4.73–44.45, 4.25–29.36, 2.25–15.07, and 2.32–12.18, respectively, which were similar to the results of Chen et al. (2018) and Song et al. (2019). According to the response value, HMDZ had the strongest aroma. The strong response of R7 and R9 sensors indicates that HMDZ contains terpenes and sulfides. Moreover, the response of R1 (benzene and structural analogues) was only found in HMDZ, which might be caused by the aromatic compounds in HMDZ. The responses of R7, R9, and R2 sensors to HZ and JZ were similar. The response values of all sensors to WJ1 and WJ2 were low, and there was no difference between them (*p* > .05), which indicated that the aroma of WJ1 and WJ2 was similar. However, it is not convincing to identify different jujube varieties by determining the response values. Therefore, PCA and HCA analyses were performed.

#### 3.2.2 Analysis of multivariate data of E‐nose

**FIGURE 4:**
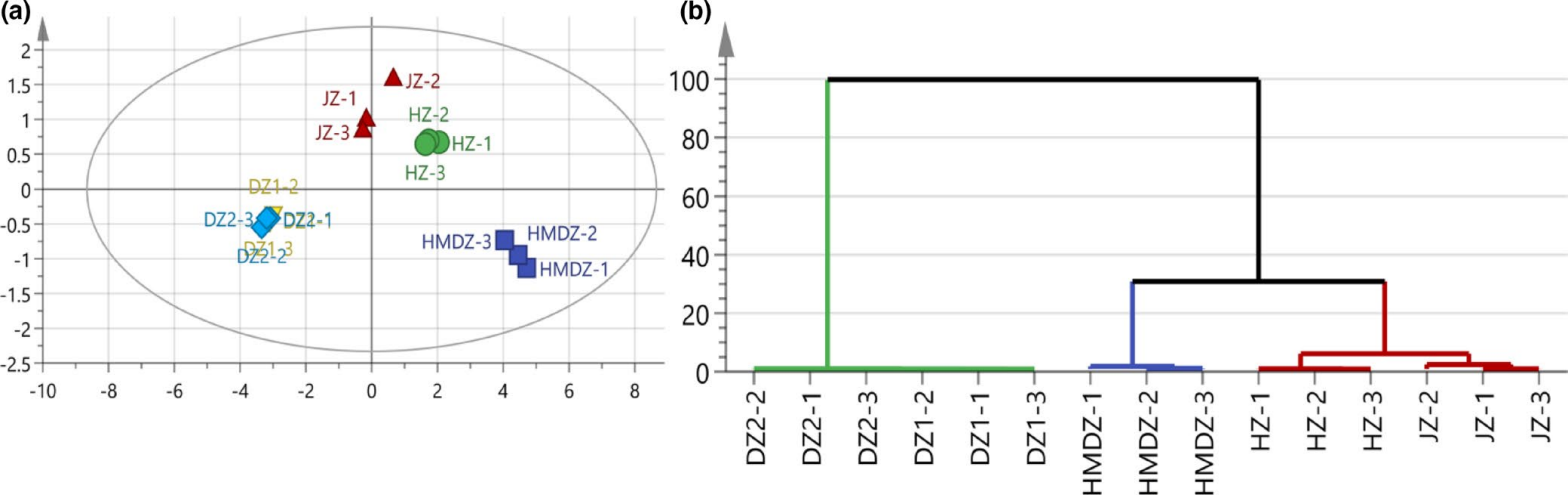
E‐nose responses to the volatile components of different jujube varieties. (a) PCA analysis of E‐nose responses to the volatile components of different jujube varieties; (b) HCA analysis of E‐nose responses to the volatile components of different jujube varieties

PCA and HCA were used to classify the response values in E‐nose analysis. The PC1 (91.10%) and PC2 (6.97%) could explain 98.07% of the variation (Figure 4a). This means that PCA analysis can effectively distinguish the volatile characteristics of different varieties. The PC1 of JZ was the highest, followed by that of HZ, HMDZ, WJ1, and WJ2. The PC2 of HMDZ was the highest, followed by that of HZ, WJ1, WJ2, and JZ. To verify the results of PCA, HCA analysis was performed (Figure 4b). The Euclidean distance (D) of 30 indicates significant separation between samples. According to the results of PCA and HCA analysis, JZ and HZ were divided into one group. There was no difference in the results of E‐nose analysis between WJ1 and WJ2, so the distribution of WJ1 and WJ2 was almost overlapped. HMDZ was separated alone, indicating that the volatile components of HMDZ were significantly different from those of other jujube varieties.

### 3.3 Combining analysis of HS‐SPME‐GC/MS and E‐nose

The comparison of the results of GC/MS and E‐nose (Figures 2 and 4) showed that the classification results were consistent: (1) HMDZ, (2) HZ and JZ, and (3) WJ1 and WJ2. Among the sensors, R7 and R9 had stronger responses, indicating that jujube fruit might contain a variety of terpenes and aromatic components (or structural analogues). The responding volatile components were identified by GC/MS. For example, 1‐penten‐3‐one, 2‐octenal, (E)‐2‐heptanal, and (E)‐2‐hexenal (terpenes and structural analogues) might cause the response of R7 (Chen et al., 2018). The response of R9 to HMDZ was the strongest, followed by that to JZ and HZ. According to the quantitative results, it was speculated that the response of R9 (sensitive to aromatic substances) might be due to the volatile components such as benzaldehyde, benzoic acid, and methyl benzoate, and the high response value to HMDZ might be due to benzaldehyde (239.2583 μg/kg). In addition, it was noticed that R6 (broadly sensitive to methane) also responded, which might be due to the volatile components containing methyl such as methyl valerate, methyl hexanoate, methyl decanoate, ethyl benzoate, methyl laurate, acetoin, and 3‐octanone.

## 4 CONCLUSION

In this study, a total of 45 kinds of volatile components were identified from the fruit of five jujube varieties (JZ, HZ, HMDZ, WJ1, and WJ2) cultivated in Xinjiang, China, and quantified by HS‐SPME‐GC/MS and E‐nose. HMDZ has the most abundant volatile components, and aldehydes and acids are the main volatile components. WJ1 and WJ2 have few differences in volatile components, which may be due to that they belongs to the same variety. Moreover, their volatile components are similar to those of HMDZ, and acids and aldehydes are the main components. In HZ and JZ, acids are the main volatile components, and the concentration is almost twice that of aldehydes. This study provides guidance for the selection of raw materials and jujube fruit processing. It also verifies that HS‐SPME‐GC/MS and E‐nose technology can quickly and accurately identify the flavor differences among jujube varieties.

## CONFLICTS OF INTEREST

The authors have no affiliation with any organization with a direct or indirect financial interest in the subject matter discussed in the manuscript.

## AUTHOR CONTRIBUTIONS

**Yuxing Liu:** Conceptualization (equal); Formal analysis (equal); Investigation (equal); Writing‐original draft (equal). **Yueying Sang:** Conceptualization (equal); Formal analysis (equal); Investigation (equal); Writing‐original draft (equal). **Jingyu Guo:** Investigation (equal); Writing‐review & editing (equal). **Weida Zhang:** Investigation (equal); Writing‐review & editing (equal). **Tianyu Zhang:** Methodology (equal); Writing‐review & editing (equal). **Hai Wang:** Formal analysis (equal); Methodology (equal); Writing‐review & editing (equal). **Shaobo Cheng:** Conceptualization (equal); Funding acquisition (equal); Project administration (equal); Resources (equal); Supervision (equal); Writing‐review & editing (equal). **Guogang Chen:** Conceptualization (equal); Funding acquisition (equal); Project administration (equal); Resources (equal); Supervision (equal); Writing‐review & editing (equal).
